# Survivability Prediction of Colorectal Cancer Patients: A System with Evolving Features for Continuous Improvement

**DOI:** 10.3390/s18092983

**Published:** 2018-09-06

**Authors:** Tiago Oliveira, Ana Silva, Ken Satoh, Vicente Julian, Pedro Leão, Paulo Novais

**Affiliations:** 1 National Institute of Informatics, Tokyo 100-0003, Japan; ksatoh@nii.ac.jp; 2 Algoritmi Centre/Department of Informatic, University of Minho, 4710-057 Braga, Portugal; silva.anapp@gmail.com (A.S.); pjon@di.uminho.pt (P.N.); 3 Department of Systems and Computation, Universitat Politécnica de València, Valencia 46022, Spain; vinglada@dsic.upv.es; 4 ICVS/3B’s, University of Minho, 4710-057 Braga, Portugal; pedroleao@med.uminho.pt

**Keywords:** survivability prediction, clinical decision support, machine learning

## Abstract

Prediction in health care is closely related with the decision-making process. On the one hand, accurate survivability prediction can help physicians decide between palliative care or other practice for a patient. On the other hand, the notion of remaining lifetime can be an incentive for patients to live a fuller and more fulfilling life. This work presents a pipeline for the development of survivability prediction models and a system that provides survivability predictions for years one to five after the treatment of patients with colon or rectal cancer. The functionalities of the system are made available through a tool that balances the number of necessary inputs and prediction performance. It is mobile-friendly and facilitates the access of health care professionals to an instrument capable of enriching their practice and improving outcomes. The performance of survivability models was compared with other existing works in the literature and found to be an improvement over the current state of the art. The underlying system is capable of recalculating its prediction models upon the addition of new data, continuously evolving as time passes.

## 1. Introduction

Colorectal cancer occurs in the lower part of the digestive tract, when growths start appearing in the walls of these portions of the large intestine. This medical condition can be further divided according to the site where the pathology develops. Colon and rectal cancers are, in fact, different pathologies, with different associated genetic causes and different progressions according to distinct molecular pathways. However, the fact of the matter is that statistics for both conditions are frequently presented jointly. About 70% of colorectal cancer cases occur in the colon and 30% occur in the rectum [1].

The severity of the cancer is determined by a process of cancer staging, consisting of determining how widespread a cancer is, how much cancer is in the organism, and where it is located [1]. Staging is performed for diagnostic and research purposes and to support the physician in planning the appropriate treatment. One of the most widely used cancer staging systems is the TNM (for tumors/nodes/metastases) system, from the American Joint Committee on Cancer (AJCC) [2]. The accurate prediction of survivability in patients with cancer remains a challenge, namely due to the heterogeneity and complexity of the disease. In cancer survivability prediction, it is important to help clinicians make the best decisions, when palliative care is an essential component of the process. The response of health care professionals to a disease largely depends on their ability to anticipate the evolution of patients [3]. Survival statistics indeed help oncologists in this task, but they are based on large groups of individuals. They cannot be used to predict exactly what will happen to a specific patient. The Kaplan–Meier method is one of the most frequently used in the conventional analysis of survivability problems [4]. It is the simplest way of computing survivability over time. This estimator can be calculated for two groups of subjects and involves computing probabilities of occurrence of an event (death) at a certain point in time. Despite the high incidence of colon and rectal cancer, there are few tools that provide survivability predictions of patients suffering from these diseases. Moreover, each tool is only capable of providing a prediction for either colon or rectal cancer, but not both.

This work is the follow-up to previous work presented in [5] and its original contributions are as follows. It describes a pipeline from data pre-processing to modeling and evaluation that enables the development of classification survivability models for 1, 2, 3, 4, and 5 years after diagnosis and treatment, for colon and rectal cancers. Treating survivability as classification problem is a different approach from the usual techniques used in this domain. Current approaches show relatively low performance, while the models presented herein show a significant improvement in this regard. The data used in the work belongs to the Surveillance, Epidemiology, and End Results (SEER) program [6], a large cancer registry in the United States, and arguably the most complete cancer database in the world. The dataset includes records of patients diagnosed with different types of cancer from 1973 to 2012. Additionally, the paper features a comparison of models based on a set of expert selected features (18 attributes) with models based on fewer automatically selected features (one third, 6 attributes). This was an objective in order to establish that models with fewer features show a close performance to those using a larger set. This is important as it is an advantage in the development of survivability calculators, with fewer inputs, deployed at the point of care. Finally, the last contribution is a system that makes the developed models available, through a mobile tool and featuring an online learning component, which no current systems possess.

## 2. Related Work

Table 1 and Table 2 show a summary of existing models for survivability prediction of colon and rectal cancers respectively and their main features. The C-index corresponds to the probability of giving a correct response in a binary prediction problem. It is considered to be numerically equivalent to the area under the ROC curve (AUC) [7]. A value of 1 represents a perfect model, whereas a value of 0.5 indicates a randomly guessing model. Most of the existing approaches for colon cancer survivability prediction are based on the SEER data. An example and one of the most widely known tools is the web-based calculator developed by Bush and Michaelson [8], whose underlying prediction model is the Nodes + Prognostic Factors (NAP), based on the number of positive lymphatic nodes combined with other prognostic features. The model has an underlying biological motivation, reflected in the use of the probability of a cancerous cell invading healthy tissues to formulate equations for cancer lethality, combined with other prognostic features estimated by means of simulation of several statistical tests. The model requires inputs for nine features and provides a prediction of the mortality risk over the period of 15 years. From the side of rectal cancer survivability prediction, it is possible to highlight the work of Valentini et al. [9], featuring an interactive tool to make survivability predictions for rectal cancer patients. The predictions are based on multivariate nomograms developed with Cox regression. They were constructed using 2795 individual patient data collected from five European randomized trials that tested preoperative chemoradiotherapy against preoperative radiotherapy.

There is a disparity in the number of features used in each tool. However, 12 [10] or even 9 [8,9] features may be too much information for a physician to introduce on-the-fly. Furthermore, there are cases in which an increased number of features does not necessarily translate into a better performance, as can be seen in the direct comparison between the works of Weiser et al. [11] and Renfro et al. [10].

Most prediction models are based on statistical modeling, namely on Cox regression analysis. This method corresponds to a multiple linear regression of the hazard on a set of variables. The discriminative power of machine learning may help to identify nuances in the data that are dismissed by statistical modeling, resulting in better performing models [16].

Al-Bahrani et al. [13], featured in Table 1, developed a survival prediction model for colon cancer, using ensemble machine learning. In this work, supervised classification methods were used to predict survivability of patients after 1 year, 2 years, and 5 years of diagnosis. SEER data from 1973 to 2009 was analyzed and passed for a cleanup process, in a total of 105,133 records. An ensemble Voting model was the one selected, with a predictive accuracy of 90.38%, 88.01%, and 85.13% for 1 year, 2 years, and 5 years, respectively, and AUCs of 0.96, 0.95, and 0.92 for 1 year, 2 years, and 5 years. Another model was proposed by Al-Bahrani et al. in [14] based on deep neural networks. This later work used the SEER data from 1988 to 2009, including 94,167 patient records for training. The resulting neural network comprised five hidden layers and reported AUCs of 0.86, 0.87, and 0.87 for Years 1, 2, and 5. The AUC was the performance measure these works focused on and hence was used as the main benchmark point.

Considering the success of recent machine learning survivability models, the classification approach was also followed in the present work. A considerable number of target predictions, either for colon or for rectal cancer, cover a five year span [10,11,15]. Even though there are models that cover a wider time span [8,9], the five year barrier is an important goal for a colorectal cancer patient to overcome, and is used throughout clinical practice guidelines [17,18] as a turning point for follow-up procedures, in which the vigilance over the patient is lightened, and for the assessment of the recurrence risk. For this reason, the present work will also have a target prediction of five years.

Regarding the selected works, survivability studies and machine learning applications of colon cancer are not covered as much as lung or breast cancer. This tendency is even more accentuated for rectal cancer. As such, literature reporting this type of work and results is not as frequent and easy to find as for other types of cancer.

Since one of the goals of the work is to build a pipeline for the generation of survivability prediction models, it is important to consider how the data will be stored and handled. This should take into account the overall work flow and purpose of the system where models will be embedded. Typically this type of model learning and maintenance involves several operations of data transformation and dynamic addition and removal of column attributes. Therefore, an agile database system, capable of performing these transformations easily and quickly is necessary. NoSQL data models provide this agility and ease of management and offer rapid and well-defined access to instance manipulation as demonstrated and discussed in [19,20]. However, a disadvantage of the NoSQL data model is not being relational. In the present work, this aspect is not important as the data consisting of a single data set, thus removing the need for a relational model, such as the one implemented in relational database management systems (RDBMSs). At the same time, the expected transaction rate of the system is low, which is another reason for excluding RDBMs. Additionally, the NoSQL data storage model can use structures such as the JavaScript Object Notation (JSON) [21], which is a lightweight data-interchange format, easy for humans to read and write, and easy for machines to parse. This kind of format is widely used to exchange information between client and server; moreover, having a database that stores these data in their original form facilitates, it removes complexity and saves time in read/write operations. In the context of the current work, this fits the intended design for the survivability prediction system.

## 3. Materials and Methods

The objective of a prediction model is to provide a forecast for the unfolding of a situation within a target label. It should be able to accept a certain number of inputs and, based on them, produce a prediction in the form of a value for the label, along with a confidence value. In this case, the output is a statement on whether a patient will survive each of the five years following treatment. The survivability prediction was handled as a classification problem. As such, five classification models for each year were developed, for both colon and rectal cancers. Each classification label (there were five representing Years 1, 2, 3, 4, and 5) could only have two values: *survived* or *did not survive*. As such, it was necessary to build five survivability prediction models (one per year) for each type of cancer. In order to provide a prediction for each year with a single interaction, the models were posteriorly programmatically combined. The development of these prediction models involved the following phases: Pre-Processing, Split Dataset, Balancing Data, Attribute Selection, and Evaluation. RapidMiner was chosen to develop the prediction models as it provides an easy-to-use application programming interface (API) that facilitates the integration of the learned models in a computational system.

### 3.1. Pre-Processing, Split Dataset, and Balancing Data

The colorectal cancer data from SEER contained 515,791 records and 146 features. During the Pre-Processing phase, the defined period of interest was from 2004 onwards, minimizing the occurrence of missing data due to the applicability of the attributes. Some of the attributes in the dataset only started to be applied after 2004, which makes the records from this period harder to compare to older records. Additionally, empty attributes, attributes that are not applicable to this type of cancer (e.g., the human epidermal growth factor receptor 2 result, an indicator used in breast cancer only) and attributes that are not directly related with the vital status of the patient were removed (e.g., the number identifying the registry of the patient). To further select the records for learning the prediction models, the following criteria were used:

- Only the adult patients (age greater than or equal to 18 years old by the time of diagnosis) were selected for further processing, as colon and rectal cancers are conditions that eminently affect the adult population and are extremely rare in individuals under 18.
- Patients who were alive at the end of the data collection, whose survivability time had not yet reached 60 months (five years), the maximum period for which the model under development is supposed to predict survivability, were excluded.
- Patients who passed away of causes other than colon or rectal cancers were excluded from the training set as their inclusion was considered to be unsuitable for the problem at hand.

After removing the records that were considered to be unfit for the survivability analysis, new labels were created in the dataset. These labels were 1-, 2-, 3-, 4-, and 5-year. According to the number of months the patients survived, the values *survived* and *did not survive* were derived for these five target labels. Finally, based on the existing attributes and at the request of the physician collaborating in this work, new attributes, such as the number of regional negative lymph nodes (extracted lymph nodes identified as not having cancer), the ratio of positive lymph nodes (having cancer) over the total examined nodes, and the relapse of the patients for colon and rectal cancer (which indicates if the patient developed cancer in the same location again), were derived.

After the Pre-Processing phase, the number of attributes in the dataset was reduced to 61, including the new attributes and the target labels. The number of records was reduced to 51,410. In the SEER data, the attributes were already in nominal form, so no conversion was needed in order to use them in the models. Each attribute had a numerical code corresponding to a category. The records were then split into two new datasets, one consisting of 38,592 records for colon cancer and another with 12,818 records for rectal cancer. In order to build training datasets, 10% of records from each dataset were randomly selected for testing and the remaining were used to develop the prediction models. After filtering records with *unknown* values, the colon cancer testing set had 2221 records and the training set had 20,061 records. The testing set for rectal cancer had 551 records and the training set had 4962 records.

Each training set was split into five sub-datasets according to the five target labels, during the Split Dataset phase. Table 3 shows the class distribution of each sub-dataset for colon and rectal cancer.

By observing Table 3, it is possible to see that the classes are not equally represented in each sub-dataset. The work in [22] dwells on the issues of using imbalanced datasets, from both the algorithmic and performance perspectives. In an overview of classification algorithms for the resolution of this kind of problem [23], it was concluded that hybrid sampling techniques, i.e., combining oversampling of the minority class with undersampling of the majority class, produce better results in knowledge extraction than oversampling or undersampling alone. As such, in the Balancing Data phase, hybrid sampling was applied in order to generate balanced sub-datasets, as described in [23]. It resulted in five sub-datasets for colon cancer with 20,061 records each and five sub-datasets for rectal cancer with 4962 records each. Each sub-dataset ends up with as many records as it started, but with a different distribution of the classes in the target label. The modeling process was carried out with balanced training datasets resulting from hybrid sampling and with the original imbalanced sub-datasets as a way to ascertain if hybrid sampling improved classification performance.

### 3.2. Attribute Selection

In order to determine the most relevant attributes for survivability prediction, it was necessary to undergo an Attribute Selection phase. This was achieved using the Optimize Selection operator [24] of RapidMiner on the five balanced sub-datasets. It implements a deterministic and optimized selection process with decision trees and forward selection. The method consists of adding variables to the model one at a time. At each step, each variable that is not already in the model is tested for inclusion. The most significant of these variables is added to the model. In this case, the limit number of attributes for inclusion was set to six. This value is one third of the expert selected features). The process was applied to each sub-dataset for the target label, for both the colon and rectal cancer. Out of the retrieved attributes for each sub-dataset, the attributes selected to make the prediction models were the ones common to all of the colon cancer sub-datasets (to build the colon cancer models) and to all of the rectal cancer sub-datasets (to build the rectal cancer models). Table 4 and Table 5 show the selected attributes and their respective meaning.

Following the attribute selection criteria, a total of six were retrieved for both colon and rectal cancer. These attributes were compared with a set of 18 attributes indicated by an expert physician, who is also one of the authors of the present paper, described in Table 6. As can be seen, for colon cancer, three of the selected attributes (age at diagnosis, AJCC stage, and regional nodes examined) were in the 18 attributes specified by the expert physician. For rectal cancer, there were four common attributes (age at diagnosis, extension of the tumor, AJCC stage, surgery of primary site, and gender). Newly added attributes, namely regional nodes negative, regional nodes ratio, and relapse, were not selected for the six attribute models, even though they were considered to be of great importance. There are cases in which, although the attribute was not selected, it is strongly related to one that was. Such is the case of the grade of the tumor, whose information is included in the AJCC stage. Except for the carcinoembryonic antigen and the CS site specific factor 2 (both selected for colon cancer), all of the remaining automatically selected attributes are closely related to the ones specified by the expert physician, which means that the attribute selection phase was able to mirror the expertise of the physician for the most part.

### 3.3. Modeling and Evaluation

The training sub-datasets for colon and rectal cancer with their respective selected attributes were used in the learning of multiple prediction models using different machine learning ensemble methods. The imbalanced training sub-datasets were also used to learn survivability prediction models. The classification schemes applied were meta-classifiers in order to boost performance. All the possible combinations of the classifiers were explored, according to the algorithms and type of attributes allowed. The tested meta-classifiers were: Bagging [25], AdaBoost [26], Bayesian Boosting [24], Stacking [27], and Voting [28]. The basic classifiers used in combination with the meta-classifiers were: k-NN (Lazy Modeling), Naive Bayes (Bayesian Modeling), Decision Tree (Tree Induction), and Random Forest (Tree Induction). Combining the meta-classifiers with the basic classifiers originated 14 different classification schemes which were applied to each sub-dataset (1-, 2-, 3-, 4-, and 5-year), for colon and rectal cancers, for hybrid sampling datasets and imbalanced datasets, and for 18 and six attributes. The performance of these classification schemes was assessed with AUC, accuracy, and F-measure. The accuracy is the percentage of correct responses among the examined cases [29]. The F-measure is a combination of precision (also known as positive predictive value) and recall (also known as sensitivity) [30]. The AUC can be interpreted as the percentage of randomly drawn data pairs of individuals that have been accurately classified in the two populations [7]. Considering the concepts of true positive (TP), false negative (FN), true negative (TN), and false positive (FP), accuracy and F-measure are defined as Equations (1) and (2).

(1) $$accuracy=\frac{TP+TN}{TP+TN+FP+FN}$$

(2) $$\begin{array}{ll} F-measure & =2\frac{\left(precision*recall\right)}{\left(precision+recall\right)}\\ & =2\frac{\left(\frac{TP}{TP+FP}*\frac{TP}{TP+FN}\right)}{\left(\frac{TP}{TP+FP}+\frac{TP}{TP+FN}\right)} \end{array}$$

These measures were calculated using the training sub-datasets and 10-fold cross validation. By applying the testing sets to the models, we calculated the percentage of incorrectly classified cases.

## 4. Experimental Results

**Figure 1:**
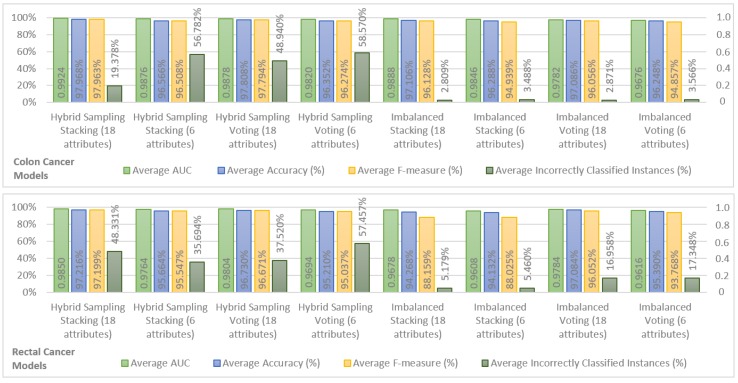
Survivability models for colon and rectal cancer for both balanced and imbalanced datasets.

In order to show the most important results, we chose the top two models in terms of performance for hybrid sampling and imbalanced datasets, in colon and rectal cancer. The results are shown in Figure 1. For the sake of brevity, only the average performance of the models for Years 1, 2, 3, 4, and 5 is shown for AUC, accuracy, F-measure, and incorrectly classified instances. It is possible to observe that both for hybrid sampling and imbalanced datasets, in colon and rectal cancers, the top two ensemble models were Stacking (using k-NN, Decision Tree, and Random Forest classifiers as base learners and a Naive Bayes classifier as a stacking model learner) and Voting (using k-NN, Decision Tree, and Random Forest as inner classifiers). In terms of AUC, accuracy, and F-measure, the performance of colon cancer survivability models was superior to the rectal cancer models, a fact that may be attributed to the fewer records available for learning the rectal cancer models.

The behavior of the different models is similar in both colon and rectal cancer, with the hybrid sampling models achieving higher performances in AUC, accuracy, and F-measure. Within these, the models using hybrid sampling Stacking with 18 attributes show the best overall performance and the models using hybrid sampling Stacking are also the best models using 6 attributes. As such, in the SEER data, the Stacking classification scheme seems to achieve better results than the Voting scheme. The downside of the hybrid sampling models is that they produce a high percentage of incorrectly classified instances in the testing sets. Comparing the percentage of incorrectly classified instances between the hybrid sampling models and the imbalanced models yields that the last produce significantly fewer incorrectly classified instances while having results that are close to the first in the remaining performance measures. Therefore, the imbalanced models can be considered to have the best compromise between the performance measures obtained from cross validation and from the testing set. Looking at these models, it is also possible to see that the difference in performance between the imbalanced models using 18 attributes and those using 6 attributes is not large, indicating that the last offer a good approximation to the 18 attribute models and are suitable for a prediction system that requires fewer inputs.

Since the objective was to find the a balance between number of inputs and predictive performance, the colon cancer model using imbalanced Stacking with six attributes was selected as a reference model. For rectal cancer, the choice is less obvious. When it comes to the imbalanced models using six attributes, the Voting model shows better accuracy and F-measure than the Stacking model. However, the Stacking model has a higher AUC and a significantly lower percentage of incorrectly classified instances, and it was thus considered that this model would perform better in unseen data and should be taken as a reference. Additional results concerting these two models, relevant for comparison with related work in the following section, are provided in Table 7.

## 5. Analysis and Discussion of Results

The number of attributes used for the survivability predictions of the reference models was six in both colon and rectal cancers. Although there are different attributes for the two types of cancer, the models require lesser attributes than the models described in [8,10,13,14] (for colon cancer), and [9] (for rectal cancer). This is an important characteristic since a long list of attributes poses an obstacle to the adoption of an application conveying the system’s functionalities. In this sense, the initial goal of developing survivability prediction models with one third of the attributes advised by an expert physician can be considered to have been achieved. Having established that this is possible, it becomes important to discover what is perceived by health care professionals as being the ideal number of input attributes for a prediction tool. This is one of the next steps in the development of the survivability prediction system.

Al-bahrani et al. in [13] use 13 attributes to produce survivability models for Years 1, 2, and 5. Their approach is similar to ours, in the sense that they also used ensemble machine learning to develop overall survivability prediction models, but just for colon cancer. The best reported model of colon cancer survival prediction was based on a Voting classification scheme, with prediction accuracies of 90.38%, 88.01%, and 85.13% and AUCs of 0.96, 0.95, and 0.92 for Years 1, 2, and 5. The reference model for colon cancer survivability prediction in our approach (imbalanced Stacking with six attributes) from Figure 1 was able to improve on these results with less than half the number of attributes. The performance values, shown in Table 7, for each year were higher, with 95.660%, 96.200%, and 97.450% of accuracy and 0.980, 0.984, and 0.985 of AUC for Years 1, 2, and 5. Our Stacking model was built with a training set consisting of 20,061 records, less than the 105,133 records used in [13]. The difference in performance might be due to the different classification attributes. Al-bahrani et al. [13] use data from the whole period of data collection, from 1973 to 2009, which implies that an important indicator such as the strict AJCC Stage would not be present in the selected attributes, as its usage started from 2004. This is a possible explanation for the results given the detailed description that the AJCC Stage offers about the state of a patient. Another comparison can be made with the deep neural network model developed by Al-bahrani et al. in [14] with respect to the obtained AUCs, namely 0.86, 0.87, and 0.87 for Years 1, 2, and 5. These results are, in fact, inferior to those of their previous work, and our approach outperforms them as well. Again, a possible explanation could be the inclusion/exclusion of the strict AJCC Stage from the selected attributes.

A loose comparison with the C-indexes reported in Table 1 and Table 2 is also possible. For the reference colon cancer survivability model, the obtained AUCs were superior to the C-index of the model by Chang et al. [12], which was the highest. For the selected rectal cancer model, the obtained AUCs shown in Table 7 were also higher than the C-indexes of Wang et al. [15] and Valentini et al. [9].

In terms of survivability prediction period, only Bush and Michaelson [8] and Chang et al. [12] offer a longer coverage in the domain of colon cancer. The first covers up to 15 years and the second up to nine years. In the domain of rectal cancer, only Valentini et al. [9] offer a longer prediction. Another limitation of our approach is the lack of a conditional survivability prediction, offered in other works such as Chang et al. [12] and Wang et al. [15].

The imbalanced Stacking models with six attributes offered a good approximation to the 18 attribute models. Therefore, they were considered suitable for a prediction system that requires fewer inputs.

## 6. CRCPredictor: A System for Cancer Survivability Prediction

Systems based on service-oriented architectures strengthen interoperability while reducing the need to synchronize data across isolated systems. At the same time, the use of mobile applications has become a common place in health care settings and provides benefits for health care professionals, namely the increased access to point-of-care tools, such as aids for disease diagnosis and medical calculators [31] that support decision-making. Considering the positive of service-oriented architectures and mobile computing aspects, this work aims to take advantage of these resources and provide a system that helps physicians improve their decisions regarding colon and rectal cancer patients. The problem it addresses is individualized survivability prediction.

### 6.1. Architecture

**Figure 2:**
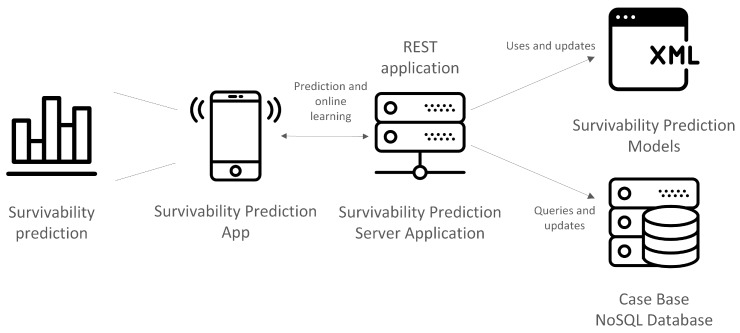
Architecture of the CRCPredictor system.

The architecture of the CRCPredictor system is shown in Figure 2. It has two main components, the Survivability Prediction App, which is a hybrid mobile application for smartphones and tablets, and a *Survivability Prediction Server Application*, which is the back-end of the system, responsible for calculating the survivability predictions and for recalculating the survivability prediction models. The application is currently available in the Google Play Store (EU and Japan) under the name CRCPredictor. It is still being optimized, so it may experience some periods of service unavailability.

The implemented architecture is service-oriented as this type offers a number of advantages, among which the most important are reusability and flexibility [32,33]. It allows the core functionality of survivability prediction to be implemented and reused in any third-party tools. Additionally, applications that consume the available web services can be written in any language, independently of the core server. We believe this may increase the reach and dissemination of the models, spawning many differently oriented applications, such as various implementations of clinical decision support systems.

The Survivability Prediction App was developed using a hybrid approach, between a web and a mobile application (for both Android and iOS). It allows the users to select the modality of prediction they intend and obtain said predictions in an effective way. The *Survivability Prediction Server Application* follows a service-oriented architecture based on RESTful web services. The choice of this type of web service in detriment of others, such as the Simple Object Access Protocol (SOAP), was motivated by the need for language and platform agnosticism, conceptual simplicity, and ease of development [34]. The available web services perform two very distinct tasks. The first group is responsible for providing survivability predictions and was developed to cover the need for an individualized system, able to respond according to a particular set of patient characteristics. This group of RESTful web services uses the RapidMiner API receives the values for the attributes and forwards them to the corresponding models, exported and encoded in XML files. The response with the survivability predictions for the five years is returned in a JSON format. The second group of RESTful web services is responsible for handling newly submitted data. They are used when health care professionals want to add new records of patients whose outcomes they already know to the *Case Base*. The submitted data concerns the attributes already selected for colon or rectal cancer, (shown in Table 4 and Table 5), and the outcomes in terms of survivability times. The data is inserted into a NoSQL database, which in this case is MongoDB. This choice was motivated by the high level of integration of MongoDB with the RapidMiner API and the fulfillment of requirements discussed in Section 2 [35]. After several insertions (currently the system is set to detect a 10% increase in data size), the models are recalculated for the type of cancer that just saw its data increase 10% (using the Stacking scheme for six attributes). In this way, it is possible to dynamically feed new cases to the prediction system and make it evolve to provide better survivability predictions. The 10% mark was arbitrarily defined and can be subject to adjustment. This part of the system uses the RapidMiner API and the designed pipeline for the modeling phase in order to generate the new models. The purpose of this is to allow the system to continuously improve with minimal human intervention. This second group of web services constitute the evolving features of the system. The system was developed with the help of a health care professional who is one of the authors. Notwithstanding the difficulties of introducing such a system in a real setting, it is one of our objectives to carry out a retrospective evaluation of the predictions provided by the system for real patients.

### 6.2. Case Example

**Figure 3:**
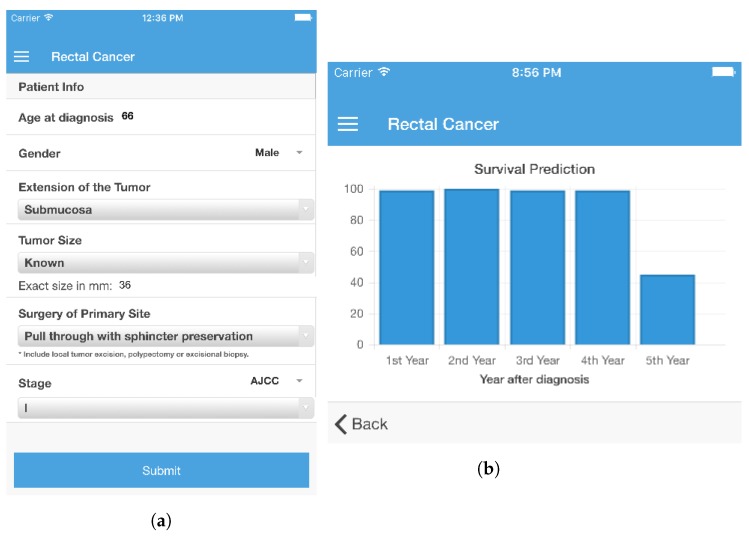
Rectal cancer survivability calculator (smartphone view). (**a**) Survivability Features of Rectal Cancer; (**b**) Results for Rectal Cancer.

In order to understand the type of interaction that a user may have with the system, the present section shows an example of a prediction. A typical use case involves an overall survivability prediction for rectal cancer. If a physician is treating a patient diagnosed with rectal cancer, once the survivability calculators are selected in the menu for the the type of cancer in question, the health care professional inserts the values for the selected attributes (Figure 3a). All attributes, except for the age of the patient, are filled in by choosing the value from a list of available options. One patient case was submitted. He was 66 years old, male, his tumor extension was in the *submucosa*, and tumor size was 36 mm. The surgery of primary site for this patient was a *pull through with sphincter preservation* and he had *stage I* cancer, according to the AJCC staging system. The values were sent to the *Survivability Prediction Server Application* and the outcome was calculated. The prediction is always provided in the form of confidence values for a positive prediction, i.e., the confidence that the patient will survive. The resulting prediction is displayed on a new screen in the form of a bar chart (Figure 3b). In this case, the models predict with 100% confidence that the patient will survive the first four years, but the prediction that the patient will survive the fifth year is just slightly above 40%. This prediction may be an indication for the physician to make arrangements for palliative care.

### 6.3. Analysis

Regarding the CRCPredictor system, it fulfills the requirements defined at the beginning of the work. The distinguishing features of the system’s architecture are its flexibility and scalability, which make the addition of new features (services) simple and easy. The Survivability Prediction App was developed as a mobile-friendly application, enabling the easy access of health care professionals to its functionalities on their mobile devices. Another component that distinguishes this system from established tools is the *Online Learning Server Application*, which ensures the continuous evolution of the prediction models without the involvement of any elements other than the health care professional.

## 7. Conclusions and Future Work

The main contribution of this work is a survivability prediction system for colon and rectal cancers. It establishes a compromise between the number of necessary inputs and prediction performance, being mobile-friendly, and featuring an online learning component that enables the automatic recalculation and evolution of the prediction models upon the addition of new cases. The developed models were able to present an overall better performance than the existing approaches. The goal with this system is to facilitate the access of health care professionals to instruments capable of enriching their practice and improving their results. In this case, rather than referring to increases in survivability times, the predictions should be used to direct the patient to the appropriate follow-up in terms of treatment options. As future work, we intend to conduct experiments to assess how well the system fulfills the needs of health care professionals and identify aspects to improve. For this purpose, the ideal setting would be a retrospective study in a health care institution with a direct comparison of predictions made by a physician, the predictions made by the system, and predictions made by other available tools.

## Figures and Tables

**Table 1:** Characteristics of models for colon and rectal cancer survivability prediction.

Characteristics	Bush and Michaelson (2009) [8]	Chang et al. (2009) [12]	Weiser et al. (2011) [11]	Renfro et al. (2014) [10]	Al-bahrani et al. (2013) [13]	Al-bahrani et al. (2017) [14]
(1) Cancer Type	colon	colon	colon	colon	colon	colon
(2) Number of Features	9	6 ${}^{‡}$	2/3/7	12	13	15
(3) Dataset	SEER	SEER	SEER	Adjuvant Colon Cancer End Points (ACCENT)	SEER	SEER
(4) Model	regression based	regression-based	regression-based	regression-based	classification-based	classification-based
(5) Target	0–15 years	1–10 years (disease specific survivability) 0–5 years (conditional survivability)	5 years	5 years	1, 2, 5 years	1, 2, 5 years
(6) Performance C-index/AUC	–	C-index: 0.816	C-index: 0.61/0.63/0.68	C-index: 0.66	AUC: 0.96/0.95/0.92	AUC: 0.86/0.87/0.87

${}^{‡}$ Including months which the patient has already survived (for conditional survivability calculation).

**Table 2:** Characteristics of models for rectal cancer survivability prediction.

Characteristics	Wang et al. (2011) [15]	Valentini et al. (2011) [9]
(1) Cancer Type	rectal	rectal
(2) Number of Features	5 ${}^{‡}$	9
(3) Dataset	SEER	five European randomized trials
(4) Model	regression-based	regression-based
(5) Target	0–5 years	1–10 years
(6) Performance C-index/AUC	C-index: 0.75	C-index: 0.70

${}^{‡}$ Including months which the patient has already survived (for conditional survivability calculation).

**Table 3:** Class distribution for each colon and rectal cancer sub-datasets according to target label.

	**Colon Cancer Target Labels**				
	**1-Year**	**2-Year**	**3-Year**	**4-Year**	**5-Year**
Did not Survive Survived	24.51%	32.60%	36.96%	39.35%	41.07%
	75.49%	67.40%	63.04%	60.65%	58.93%
	**Rectal Cancer Target Labels**				
	**1-Year**	**2-Year**	**3-Year**	**4-Year**	**5-Year**
Did not Survive Survived	4.03%	5.89%	7.17%	8.08%	8.70%
	87.88%	82.27%	78.41%	75.68%	73,79%

**Table 4:** Attributes retrieved by attribute selection and used for colon cancer models.

Attribute	Description
Age at diagnosis	The age (in years) of the patient at time of diagnosis
Carcinoembryonic Antigen	The interpretation of the highest Carcinoembryonic Antigen test results
CS Site-Specific Factor 2	The clinically evident regional lymph nodes
AJCC Stage	The grouping of the TNM information combined from the American Joint Committee on Cancer
Primary Site	Identification of the site in which the primary tumor originated
Regional Nodes Examined	The total number of regional lymph nodes that were removed and examined by the pathologist

**Table 5:** Attributes obtained by attribute selection and used for rectal cancer models.

Attribute	Description
Age at diagnosis	*1
Extension of the Tumor	Information on extension of the tumor
Tumor Size	Information on tumor size
AJCC Stage	*1
Surgery of Primary Site	Describes a surgical procedure that removes and/or destroys tissue of the primary site performed as part of the initial work-up or first course of therapy
Gender	The sex/gender of the patient at diagnosis

*1 Described in Table 4.

**Table 6:** Attributes selected by an expert physician.

Attribute	Description
Age at Diagnosis	*1,*2
Extension of the Tumor	*2
CS Site-Specific Factor 8	The perineural Invasion
Tumor Size	*1
AJCC Stage	*1,*2
Grade	Grading and differentiation codes
Histologic Type	The microscopic composition of cells and/or tissue for a specific primary
Laterality	The side of a paired organ or side of the body on which the reportable tumor originated
Surgery of Primary Site	*2
Race Recode (White, Black, Other)	Race recode based on the race variables
Regional Nodes Examined	*1
Regional Nodes Positive	The exact number of regional lymph nodes examined by the pathologist that were found to contain metastases
Regional Nodes Negative	(Regional nodes examined - Regional nodes positive)
Regional Nodes Ratio	(Regional nodes negative over Regional nodes examined)
Relapse	The relapse of the patients for cancer
Gender	*2

*1 Described in Table 4; *2 Described in Table 5.

**Table 7:** Accuracy and AUC results for the imbalanced Stacking models with six attributes for colon and rectal cancers.

Model	Performance Measure	1-Year	2-Year	3-Year	4-Year	5-Year	Average
Imbalanced Stacking with 6 attributes (colon)	Accuracy	95.660%	96.200%	96.440%	96.690%	96.450%	96.288%
	AUC	0.980	0.984	0.986	0.988	0.985	0.9846
Imbalanced Stacking with 6 attributes (rectal)	Accuracy	94.420%	94.450%	94.050%	93.890%	94.510%	94.132%
	AUC	0.957	0.960	0.961	0.963	0.971	0.9608

## Abbreviations

The following abbreviations are used in this manuscript:

AJCC	American Joint Committee on Cancer
AUC	Area under the ROC curve
SEER	Surveillance, Epidemiology, and End Results (SEER)
